# The Preservation of Natural Teeth

**Published:** 1864-01

**Authors:** S. B. Palmer


### THE PRESERVATION OF NATURAL TEETH.

**Read before the Central N. Y. Dental Association, Nov., 1863. By S. B. Palmer, D.D.S.**

*Mr. President and Gentlemen:* When about to engage in any calling or enterprise worth our attention, the following questions should be asked, and, if possible, a satisfactory answer obtained, before proceeding by way of experiment—subject to loss of time and money by repeated failures and unsuccessful results:

1st. How important? How much real benefit can be derived from the undertaking, if successful; and what will be the result, not only to ourselves, but to those connected or associated with us?

2d. Do we possess the knowledge, skill, ambition and means, to warrant the undertaking?

As members of the dental profession, we to-day stand in nearly the same position, and should be prepared to answer similar questions.

The subject before us is of immense importance to every one; it is the cause of much thought and anxiety from youth to old age, and should contemplate the highest aim and engage unyielding perseverance from every member of the dental profession.

The first question—How valuable are natural teeth?—is the subject of this essay.

The second query—Do we possess the knowledge and skill necessary; or do we understand all that is known of the various means and agents employed to arrest disease and assist Nature, in retaining those valuable organs so intimately connected with the well-being of the human race?—remains to be answered by the members of this Association who are willing to contribute to a general fund of knowledge for the benefit of each and all.

It is not our design to present the various causes which have influenced or produced either caries or an abnormal condition of the teeth; but to take into consideration the design of Nature in forming those organs, and the importance of their preservation.

We believe that mankind was created physically sound; that each member of the organization was capable of performing the office assigned it. We can not so reflect upon the Author of our existence, as to say or believe that man was an exception in the whole animal creation, thus to differ from the lower animals, by being compelled to have portions of his frail body drawn asunder, or, by a still more tedious process, submit to time, aided by disease and decay, to effect the same object; and thus cut off one organ after another, until humanity becomes almost disorganized—"diseased—decaying, dying, and still living." Such is man.

Who will not say that man, so far as this life is concerned, pays a heavy tax over the inferior creation, for his much-boasted reason.

No—we can not charge this misery upon our Creator; and if we can not, then do we admit that the dental organs were originally perfect, as were the other organs of the body,—which again implies that by depraved habits or indulgences, and physical transgressions, this lamentable evil is entailed upon the human race.

As well might Old Time with his scythe commence clipping off the extremity of our limbs, and thus, inch by inch, dismember our bodies, as to commence, by decay, upon the frame, machinery or grinding apparatus of the dental mill, from which our bodies receive subsistence.

We may regard our physical organization as a chariot in which the soul is traveling, while on the journey of life. Sad as the thought may be, many of those chariots are weak and frail,—in constant danger of breaking down. From this fact, and to correct in some degree this evil, has the science of dentistry been developed.

Dentists and physicians are but artizans called into requisition to lift at the wheels, strengthen the weaker portions, and supply the lost parts of this chariot, that it may remain sufficiently strong to reach the journey's end.

It is a saying in mechanics, that a machine is no stronger than its weakest part. So with man; however strong and healthy, deprive him of properly-prepared food, and the result is the derangement of the whole machinery of the physical organization, which is always the case with bad teeth or imperfect mastication.

The teeth appear to be the first organs attacked by the "King of Terrors." In youth may be seen the marks of the destroyer; which rapidly increase with advancing years, until it has become a rarity to witness anything like a perfect, natural denture at an advanced age in life.

The dental profession is called upon to correct this growing evil. Various means are recommended and adopted to meet the demands.

While some of the profession are willing to admit that our Creator knew best how to make man, and are desirous to assist with all the skill they possess in correcting the evil brought upon the race by transgressions, and thus preserve the type. Others appear to have lost confidence in the skill of the Great Creator of the human family as relating to the dental organs, and devote their whole energies in the transformation of man to manikin.

We would as soon charge a surgeon with unnecessary amputations, because he was known to have an interest in an artificial-limb manufactory as to charge a respectable dentist with extracting teeth that he knew could be preserved and rendered serviceable, for the simple reason that he might obtain his fee for replacing them with artificial substitutes.

And yet with dentists there is a vast difference of opinion as to the real value of *natural* teeth; also, in their confidence of success, or willingness to undertake their preservation; when cases are presented for examination, consisting of a mere remnant of a *once* beautiful arch, with here and there a tooth standing sentry-like, surrounded by disease and attacked by decay.

While one, not understanding the diagnosis of the disease, would refuse to prescribe remedies without first removing all exciting causes, and perhaps recommend extracting sound teeth, from diseased gums. Another, who had made that branch a specialty, could, by the light of science, "see the end, from the beginning," would regard the case both simple and curable.

While one operator may adopt as his rule, the extraction of frail teeth, and those with exposed nerves; another would be prepared to offer consolation and final restoration, by the application of proper remedies and the skillful use of the plugger.

One may feel proud of his attainments and success in the treatment and preservation of natural teeth, and spend his time and energies at the operating chair; another may feel equally as proud of his artificial substitutes and the mechanical productions of his laboratory.

When a surgeon is consulted in cases of diseased or fractured limbs, to decide between preservation or amputation, to the patient such decision is of great importance; and when the ability to decide correctly is called in question, the case is referred to others.

Why so much painstaking? Because Doctors have been known to disagree.

Many of the cases presented to dentists, relating to the teeth simply, require as nice points of decision as though the same was to determine the fate of a limb; and yet so little is thought of the matter that even the most trifling circumstances turn the scale. Why is this? Because not only the community, but many dentists, undervalue natural teeth.

"The teeth of inferior animals appear to fulfill their design, by mastication, offensive and defensive operations; while with man, who is alone endowed with speech, the teeth act an important part in controlling and modulating the voice—in giving beauty and expression to the countenance—symetry, fullness and boldness of outline to the surrounding parts—that can not be obtained by artificial means."

Did every person believe that a natural tooth, when retained in the mouth in a healthy condition, was worth fifty dollars,—then would they feel that they had made a good investment, when, by the payment of two or three dollars, one such organ had been restored from decay and rendered artificially sound. When at the commencement of the rebellion, which is now measuring strength with our Government, we read that a few of our brave volunteers had fallen in defense of our country, we were pained and saddened. Strange as it may appear, by the almost weekly addition of hundreds to the list of the dead, human life appears of less value,—so that the intelligence that a hundred or even a thousand as brave as the first have fallen, we remember it but a few days and are ready for more startling news.

So with the loss of teeth, though a great misfortune, but little is thought of it, on account of its frequent occurrence and the exceeding low prices for substitutes.

The painful operation of submitting to the extraction of teeth, together with the high price of artificial dentures, caused many in former years to resort to a less painful and expensive operation,—that of preserving their natural teeth by filling.

But, since the introduction of Rubber, and with it *rubber workers*, to the profession; since people are called upon to patronize certain Doctors who offer to insert full sets of teeth for seven or eight dollars—traveling expenses and board included—natural-teeth stock went down.

And now, Gentlemen, let us beware, since the application of nitrous oxide gas as an anæsthetic, by which tooth-extracting is said to be rendered a pleasant pastime, that there is not a greater depreciation in the value of natural teeth.

We would not be understood to say that all natural teeth could, by any means, be preserved for beauty or usefulness; yet we know that many valuable ones are daily sacrificed that by skillful treatment could be retained for use.

Let us not only try to educate each other as to the best means of preserving teeth, but try and instruct our patients to understand that they also have an important part to perform for their own benefit, in the bestowal of care, cleanliness and close examination.

